# What About *Promotores*? *Promotores'* Psychosocial Determinants That Influenced the Delivery of a Cervical Cancer Education Intervention to Hispanics

**DOI:** 10.3389/fpubh.2021.689616

**Published:** 2021-09-10

**Authors:** Julie St. John, Belinda Reininger, Hector Balcazar, Melissa A. Valerio-Shewmaker, Christopher E. Beaudoin

**Affiliations:** ^1^Department of Public Health, Graduate School of Biomedical Sciences, Texas Tech University Health Sciences Center, Abilene, TX, United States; ^2^University of Texas Health Science Center at Houston School of Public Health, Brownsville, TX, United States; ^3^College of Science and Health, Charles R. Drew University of Medicine and Science, Los Angeles, CA, United States; ^4^College of Communication, Boston University, Boston, MA, United States

**Keywords:** training, Hispanics, cancer education intervention, promotores, community health worker, psychosocial determinants

## Abstract

This study tested whether a cancer education intervention affected *promotores'* self-efficacy to deliver an intervention to Hispanics and which psychosocial determinants of *promotores* influenced the number of Hispanic residents reached by *promotores* in the subsequent education intervention. A quasi-experimental, pre/post-design with a treatment group (no control) assessed differences for *promotores* (*n* = 136) before and after exposure to the cancer education intervention. The design also included a cross-sectional evaluation of the number of residents *promotores* reached with the educational intervention. After being trained, the *promotores* delivered the intervention to Hispanic residents (*n* = 1,469). Paired *t*-tests demonstrated increases in *promotores'* self-efficacy from pre- to post-intervention. Regression models assessed associations between the numbers of residents reached and select psychosocial determinants of *promotores*. Age and *promotores*' years of experience influenced their delivery of a cervical cancer education intervention to Hispanics, but not their delivery of breast or colorectal cancer education interventions. This is the first study to examine which psychosocial determinants influence *promotores* delivery of cancer education interventions. The outcomes potentially have implications for CHW interventions and training by examining this potential connection between CHWs' psychosocial determinants and intervention outcomes.

## Introduction

“*Promotores are everyday people who are already living, working and engrained in the community…They are the individuals others go to when they have problems, need advice or even just want to gossip over a cup of coffee”* (1).

As frontline public health workers, *promotores* [community health workers (CHWs)], function as liaisons between health and social service providers and the priority population to facilitate access to services and improve the quality and cultural competence of service delivery through a wide array of skillsets. *Promotores* are trusted members of the community and have a remarkable understanding of the community, may work for pay or as volunteers, and usually share ethnicity, language, socioeconomic status, and life experiences with the community members they serve (2–4). Core functions of *promotores*/CHWs have included health education, promotion, outreach, case management, service coordination, informal counseling, social support, advocacy, referrals, and health behavior interventions (5, 6). The utilization of *promotores* in the United States has grown particularly in the past 10 years (7, 8) along with evidence supporting the effectiveness of *promotores* in delivering interventions (9–11). Numerous studies have highlighted the use of *promotores* and their effectiveness among Hispanic populations in helping their priority populations achieve positive health outcomes (5, 8–11).

Training involved in preparing *promotores* to conduct interventions is substantial and comprehensive and incorporates *promotores* in decision making and feedback regarding project implementation after the training (12–14). Yet, even with intensive training, like other conduits for the delivery of health education content, there are barriers to the implementation of interventions delivered by *promotores* (15, 16). An important translational science question is what characteristics of *promotores* influence the delivery of interventions (17, 18). Proven, effective interventions involving *promotores* delivered under highly controlled settings are fruitless if, in a practice setting, there are problems with the delivery of the intervention by *promotores*. A greater understanding of what characteristics influence that delivery is needed for the more efficient diffusion of interventions led by *promotores*.

This study seeks to fill this gap by examining the psychosocial determinants of *promotores* in relation to the delivery of a cancer education intervention to Hispanic residents. Few studies have looked at psychosocial determinants of *promotores*' delivery of an intervention (19), and no studies were found that focus on these psychosocial determinants of *promotores* specific to cancer education interventions. Psychosocial determinants are defined as the interaction of psychological factors (e.g., an individual's thoughts, self-efficacy, intentions, and behaviors) and social factors (e.g., education, employment, social norms and attitudes, social support and interactions, and socioeconomic conditions) that influence health status (20, 21).

This study focused on cancer delivered interventions among Hispanic populations given that cancer is the second leading cause of death among Hispanics (22); Hispanics are less likely to obtain cancer screenings (23, 24); Hispanics are often diagnosed and treated at later stages of cancer (25–27); and Hispanics face greater cancer survivorship barriers than non-Hispanic Whites (25, 27). Evidence suggests key factors contributing to poorer cancer outcomes in Hispanics include the following: socio-demographic factors such as poverty, lack of education and information, and lack of health insurance; language barriers; and low health literacy (26, 28–30). Furthermore, this study included information on cervical cancer education intervention since Hispanic females have the highest incidence and the second highest mortality rate of cervical cancer in the United States (25, 31) and face numerous barriers regarding cervical cancer prevention and screening (31–34).

This study had two main aims: (1) to test whether a cancer education intervention affects *promotores*' self-efficacy from pre- to post-training; and (2) to examine which psychosocial determinants of *promotores* might influence how many Hispanic residents receive cancer education interventions delivered by the *promotores*. Self-efficacy is an important outcome given its role in different health behavioral theories (e.g., Health Belief Model) as an antecedent of health behavior. It can be defined in terms of people's beliefs in their capability to perform a specific behavior to achieve an anticipated outcome (35). The psychosocial determinants of *promotores* examined included *promotores*' years of work experience, work status (paid or volunteer), self-efficacy to deliver cancer education to Hispanic residents, intention to use the information in his/her work and *promotores*' certification status.

## Data and Methods

### Parent Study

The parent study—É*PICO: Education to Promote Improved Cancer Outcomes*—was a cancer education intervention evaluated by an exploratory quasi-experimental, pre-test-post-test study design. The overall strategy of É*PICO* was to train and utilize *promotores* as learners and educators to deliver a cancer education intervention to Hispanic *colonia* residents. Community Health Workers/Promotores in Texas are certified, which means they have either completed a 160-h certification training as CHWs/Promotores or they obtained certification through at least 1,000 verified hours of work as a CHW/Promotora (36). *Colonias* are unincorporated sub-divisions lacking basic infrastructure and services (37). The study consisted of three separate training modules that were 8 h in length each (one 8-h training for breast cancer prevention, treatment and survivorship; one 8-h training for cervical cancer prevention, treatment and survivorship; and one 8-h training for colorectal cancer prevention, treatment and survivorship). While the curriculum covered the continuum of cancer prevention, treatment, and survivorship, each training had specific elements and evidence-based information for each section as well as covering information related to prevention, treatment, and survivorship throughout the 8-h trainings. Promotoroes could choose to attend one, two, or all three É*PICO* trainings, provided at different times during the project period. The selection criteria for participation was self-identification as a promotora and being at least 18 years of age.

The É*PICO* cancer education interventions were grounded in the socio-ecological model, the health belief model, the stages of change (transtheoretical) model, and evidence-based principles of adult learning theory—engaging *promotores* in an interactive environment based upon discussion and skill-building exercises (38, 39). The focus groups and surveys utilized constructs from the aforementioned theories; these theories also then provided a framework for the module content. For example, from the health belief model, the training materials addressed perceived susceptibility, perceived severity, perceived benefits, perceived barriers, cues to action, and self-efficacy (40). For a detailed outline of content for each É*PICO* cancer education intervention and for more information on methods, refer to previous publications on the É*PICO* study (41, 42).

### Study Design

For the purposes of this manuscript, a quasi-experimental, pre-post, one group design was used for this exploratory study. Pre/post-tests assessed differences for *promotores* (*n* = 136) before and after exposure to the intervention on prevention, treatment, and survivorship for breast, cervical, or colorectal cancer. The design also included time-lagged evaluations of the number of residents *promotores* reached. Data were collected from *promotores* who attended an 8-h training on prevention/early detection, treatment, and healthy survivorship for breast, cervical, or colorectal cancers *via* pre/post-tests as the data collection instruments. These measures included an assessment of psychosocial determinants of the *promotores*. Data were collected from the *promotores* on the number of Hispanic residents they reached with a cancer intervention within the 2 months following their initial training. This data included intervention logs of the number of residents reached as the data collection instrument.

### Study Setting

The study setting included 8-h trainings on prevention, treatment, and healthy survivorship for breast, cervical, or colorectal cancers for self-identified *promotores* conducted in four south Texas border counties. The trainings were conducted by six state-certified *promotora* instructors employed by the É*PICO* project at community partner facilities, including the following: academic partners (four trainings—two in Hidalgo County and two in Cameron County), a community resource center (one training, Hidalgo County), and a county-owned facility (one training, Cameron County). *Promotores* who received the É*PICO promotora* cancer education intervention then delivered the interventions to Hispanic *colonia* residents in the four counties as part of their regular *promotora* outreach and education responsibilities.

### Recruitment and Procedures

Study participants included both *promotores* and Hispanic *colonia* residents. The study did not include a study size calculation. First, *promotores* serving Hispanic *colonia* residents in the four counties were recruited by emails to distribution lists, participants from previous trainings, partnering entities, the state *Promotora*/CHW Program contact list, and word of mouth. Study staff obtained informed consent, and participants filled out the pre-test questionnaire. É*PICO* certified instructors gave the 8-h trainings to the *promotores*, which covered detailed information on prevention, treatment, and healthy survivorship specific to either breast, cervical or colorectal cancers. The training included activities to ensure that the topics were learned and a review and practice time associated with the specifically designed intervention modules that were to be delivered to residents. After the training, *promotores* completed the post-test questionnaire, which included the pre-test measures and demographic and psychosocial measures. Second, within 2 months of receiving the training, *promotores* were given the option to implement the educational intervention with Hispanic *colonia* residents. The intervention for the residents consisted of the same topics—prevention, treatment, and healthy survivorship—for either breast, cervical, or colorectal cancers; the resident interventions covered broader, key points pertaining to these topics and included important facts, photos, questions and answers, and action planning. *Promotores* opting to deliver the cancer education interventions obtained consent, conducted the intervention (i.e., 1.5 h of education per cancer type with residents), collected resident collection pre/post-tests and evaluations, and returned study instruments to É*PICO* staff within 2 months of the initial É*PICO promotora* training. *Promotores* who educated at least 10 Hispanic *colonia* residents received a $25 Wal-Mart gift card. The *promotores* were not compensated through salary support for their time to provide the education to *colonia* residents.

### Measures

The pre/post-tests had 15 knowledge and six self-efficacy survey items. Self-efficacy was measured using a Likert scale (low confidence: 1–2; medium confidence: 3–4; and high confidence: 5–6) and measured participants': (1) confidence in delivering (breast, cervical, colorectal) cancer prevention/early detection messages; (2) confidence in motivating others to take steps toward (breast, cervical, colorectal) cancer prevention/early detection; (3) confidence in developing (breast, cervical, colorectal) cancer treatment messages; (4) confidence in motivating others to obtain (breast, cervical, colorectal) cancer treatment; (5) confidence in delivering (breast, cervical, colorectal) survivorship messages; and (6) confidence in motivating others to take steps toward healthy (breast, cervical, colorectal) cancer survivorship behaviors. In addition, post-test included demographic and psychosocial measures. The dependent variable was the number of *colonia* residents who received the cancer education intervention by the *promotores*. This was determined from the *promotores*' intervention logs detailing the number of residents educated by cancer education module. The number of Hispanic *colonia* residents reached by *promotores* was treated as a continuous variable. We also examined the number of *colonia* residents reached by *promotores* per cancer type (i.e., breast, cervical, or colorectal).

The independent variables were separated into two categories: psychosocial determinants of *promotores* and control variables. Psychosocial determinants of *promotores* included: (1) *promotores*' years of work experience; (2) work status (paid or volunteer); (3) self-efficacy in delivering cancer education to Hispanic *colonia* residents; (4) intention to use the information in his/her work (scale from 1 to 4 with 1 = not at all true, 2 = not true; 3 = somewhat true; and 4 = very true); and (5) certified *promotor/a* (yes/no). For *promotores*' self-efficacy, measures were recorded for each cancer type both pre- and post-training, (scale from 1 to 6, with low self-efficacy being 1 and high self-efficacy being 6). Three questions measured self-efficacy for each specific type of cancer module: (1) self-efficacy in delivering (breast, cervical, colorectal) cancer prevention/early detection messages; (2) self-efficacy in delivering (breast, cervical, colorectal) cancer treatment messages; and (3) self-efficacy in delivering (breast, cervical, colorectal) cancer survivorship messages. Cronbach alphas were used to test the reliability of the scale measuring the self-efficacy values. The self-efficacy variables for pre- and post-measures for breast, cervical, and colorectal cancers all had reliability values of α = 0.96 or higher (reported in **Table 3**).

Control variables included the following: (1) age (years of age); (2) gender (male/female); (3) education (with the following responses: some high school; high-school graduate; GED; technical degree; some college; Bachelor's degree; advanced degree); and (4) the number of cancer type trainings received by the *promotores* (with the following responses: one cancer type; two cancer types; and three cancer types).

This study was approved by the Committee for the Protection of Human Subjects (CPHS) at the University of Texas Health Science Center, and the parent study was approved by the Institutional Review Board (IRB) at Texas A&M University.

### Data Analysis

Analyses were conducted using STATA 12.0 (43). Missing values were replaced with mean scores since data were missing in <5% of any variable (44). Factor analysis and Cronbach's alphas were run on each of the sets of self-efficacy items for pre- and post-measures for breast, cervical, and colorectal cancers. Paired *t*-tests for the pre- and post-self-efficacy scores were run to test for differences among the pre- and post-self-efficacy means for the three cancers; *p*-values < 0.05 were considered significant. Regression models were run to assess potential associations between the continuous dependent and continuous and dichotomous independent variables. Three regression models (one per cancer) assessed predictors and included statistically significant variables (*p* < 0.05) related to the dependent variable.

## Results

A total of 136 *promotores* received the É*PICO promotor/a* training modules. Of those, 50% (68) delivered the cancer education intervention to 1,469 Hispanic *colonia* residents. Table 1 further details the number of residents receiving the specific cancer education interventions delivered by *promotores*. The psychosocial determinants and demographic control variables of the *promotores* who received at least one É*PICO* cancer education intervention are displayed Table 2. *Promotores* tended to be women (95%), had an average age of 48 (range of 20–73), had a high-school education or less (53%), had an average of 6.5 years of work experience as *promotores* (ranged from 0 to 25 years), and were Texas-certified *promotores* (68%). Hispanic *colonia* residents who received a cancer education invention delivered by these *promotores* included 450 residents receiving the breast cancer education intervention; 506 residents receiving the cervical cancer education intervention; and 513 residents receiving the colorectal cancer education intervention. The factor analysis followed a normal distribution, with no outliers and points randomly distributed about zero.

**Table 1:** The number of residents receiving the specific É*PICO* cancer education interventions delivered by *Promotores*.

**Cancer education intervention**	**# Of *Promotores* who received this intervention training**	**# Of *Promotores* who delivered this intervention to *colonia* residents**	**# Of *Colonia* residents who received this intervention**
Breast cancer	94	41	450
Cervical cancer	74	42	506
Colorectal cancer	81	45	513
Total numbers	136	68^*^	1,469

* *Total number of unduplicated promotores who trained colonia residents. Some promotores attending more than one ÉPICO cancer education intervention training delivered multiple cancer education interventions to colonia residents*.

**Table 2:** Psychosocial determinants and control variables of *Promotores*.

**Independent variables**	**Counts**	**Frequency**	***M***	**SD**
**Psychosocial determinants of** ***Promotores***				
*Promotores* of years of work experience	-	-	6.49	5.93
Employment status, paid				
No	52	38.2%	-	-
Yes	74	54.4%		
Missing	10	7.4%		
Employment status, volunteer				
No	89	65.4%	-	-
Yes	37	27.2%		
Missing	10	7.4%		
Breast cancer, pre-test self-efficacy	-	-	4.67	1.19
Breast cancer, post-test self-efficacy			5.50	0.79
Cervical cancer, pre-test self-efficacy			4.63	1.01
Cervical cancer, post-test self-efficacy			5.43	0.75
Colorectal cancer, pre-test self-efficacy			4.37	1.17
Colorectal cancer, post-test self-efficacy			5.53	0.63
Intention				
Not true at all	0	0%	-	-
Not true	0	0%		
Somewhat true	10	7.4%		
Very true	126	92.6%		
DSHS certified *Promotor/a*				
No	52	38.2%	-	-
Yes	84	61.8%		
Age	-	-	47.91	9.15
Gender				
Female	129	94.9%	-	-
Male	7	5.1%		
**Control variables**				
Education				
Some high school	26	19.1%	-	-
High-school graduate	17	12.5%		
GED	27	19.9%		
Technical degree	9	6.6%		
Some college	16	11.8%		
Bachelor's degree	15	11.0%		
Advanced degree	10	7.3%		
Other	16	11.8%		
Number of trainings received by *promotores*				
One	64	47.1%	-	-
Two	32	23.5%		
Three	40	29.4%		
Employing agency				
Medical clinic	8	5.9%	-	-
Hospital	0	0%		
Home health agency	11	8.0%		
Other medical entity	0	0%		
Non-profit	58	42.7%		
Social service entity	5	3.7%		
University/academic	16	11.8%		
Other	38	27.9%		

The paired *t*-test results for the pre- and post-*promotora* training self-efficacy scores on the three cancer topics are shown in Table 3. There were significant increases in *promotores'* pre- and post-training scores for each cancer specific training (*p*-values for all three cancers < 0.000).

**Table 3:** Paired *T*-test results for *Promotores'* pre- and post- training self-efficacy scores.

**Variable**	**Obs**.	**Mean**	**Std. Dev**.	**95% Conf. interval**
**Breast cancer**				
*N* = 94*, **p*** **<** **0.000**				
Self-efficacy - pre-test	94	4.67	1.19	(4.42, 4.91)
Self-efficacy - post-test	94	5.50	0.79	(5.34, 5.66)
Difference between pre and post	94	−0.83	0.91	(−1.02, −0.65)
**Cervical cancer**				
*N* = 74, ***p*** **<** **0.000**				
Self-efficacy - pre-test	74	4.63	1.01	(4.40, 4.87)
Self-efficacy - post-test	74	5.43	0.75	(5.26, 5.60)
Difference between pre and post	74	−0.80	0.76	(−0.96, −0.62)
**Colorectal cancer**				
*N* = 71, ***p*** **<** **0.000**				
Self-efficacy - pre-test	81	4.37	1.17	(4.11, 4.63)
Self-efficacy - post-test	81	5.53	0.63	(5.39, 5.67)
Difference between pre and post	81	−1.16	0.99	(−1.38, −0.94)

*Bold values are statistically significant p value < 0.000 is highlighted*.

Table 4 depicts results of the regression models examining whether there are significant psychosocial determinants associated with delivering cancer education; on breast cancer (model 1); on cervical cancer (model 2); and on colorectal cancer (model 3). For the delivery of cervical cancer education, *promotores*' years of work experience (*P* > |*t*| = 0.000), age (*P* > |*t*| = 0.003), and the number of *promotor/a* cancer trainings received (*P* > |*t*| = 0.001) were significant in the cervical cancer model, which included all of the independent and control variables. Additionally, the number of *promotor/a* cancer trainings received was also significant for breast cancer (*P* > |*t*| = 0.000) and colorectal cancer (*P* > |*t*| = 0.020). The *R*^2^-values for each module were as follows: *R*^2^ = 0.26 for breast cancer, *R*^2^ = 0.41 for cervical cancer, and *R*^2^ = 0.12 for colorectal cancer.

**Table 4:** Regression analysis for psychosocial variables predicting number of *Colonia* residents trained in cancer education (*N* = 136).

	**Model 1** ** (Breast cancer)**		**Model 2** ** (Cervical cancer)**		**Model 3** ** (Colorectal cancer)**	
**Independent variables**	**Coef**.	***t***	**Coef**.	***t***	**Coef**.	***t***
*Promotores'* years of work experience	−0.1663	−1.73	**0.4627**	**3.72**	0.0349	0.27
Employment-Paid	0.7229	0.37	−1.623	−0.53	−1.060	−0.38
Employment-volunteer	2.547	1.21	−0.0602	−0.02	−0.7504	−0.26
Self-Efficacy, post-test (breast, cervical, colorectal)	0.4585	0.64	1.409	1.31	0.4196	0.32
Intention	−0.1374	−0.06	1.200	0.70	−0.8633	−0.27
DSHS-certified	−1.850	−1.55	3.078	1.71	0.9739	0.56
Age	0.0876	1.35	**−0.2856**	**–3.07**	0.0985	1.22
Gender	3.423	1.39	3.660	1.21	−1.599	−0.41
Education	0.2600	0.85	0.7079	1.70	0.0887	0.22
Number of trainings received	**2.767**	**4.25**	**3.454**	**3.59**	**2.165**	**2.39**
Prob > *F*	**0.0037**		**0.0001**		0.4708	
*R* ^2^	0.2592		0.4087		0.1225	

*Numbers in BOLD and BLUE represent a significant P > |t| value*.

## Discussion

This study is the first of its kind to examine which psychosocial determinants of *promotores* influence their delivery of cancer education interventions to Hispanic *colonia* residents. Results showed that *promotores*' years of work experience, *promotores*' age, and the number of cancer trainings received by *promotores* were significantly associated with the number of Hispanic *colonia* residents who received the cervical cancer education intervention. This is noteworthy because this is the first study to suggest which psychosocial determinants might affect the delivery of a cancer education intervention. In light of the burden of cervical cancer incidence and mortality on Hispanic females, interventions utilizing *promotores* in cervical cancer education interventions might consider these psychosocial determinants—age, *promotores*' years of work experience, and number of other relevant trainings—when recruiting *promotores* to implement interventions. Our results also suggest that psychosocial determinants of *promotores* are associated with intervention delivery—which constitutes a novel contribution to the literature.

Another important finding was the effectiveness of the cancer education interventions to increase *promotores*' self-efficacy pre- and post-training. We found that the changes between pre- and post-self-efficacy measures were significant for all three cancers and that the self-efficacy measures were reliable. Though self-efficacy was not found to be significant in these regression models to predict the number of residents reached, this finding of the cancer education interventions might have influenced other desired outcomes not examined in this study (such as increased knowledge of the residents receiving the intervention and higher residents' intentions to change behavior). Future studies could examine how this increased self-efficacy might have influenced additional outcomes of interest.

Lastly, of note in this study is what associations between variables were not found to be significant. First, the study examined breast, cervical, and colorectal cancer education interventions for Hispanic *colonia* residents, yet, only two psychosocial determinants were found to be significant for cervical cancer. This creates questions regarding why these factors were significant for one cancer and not the others—suggesting that *promotora* psychosocial determinants influencing intervention delivery to residents may be different depending the intervention topic—whether type of cancer or specific chronic disease. Second, though the study looked at numerous *promotora* psychosocial determinants, few were found to significantly impact their intervention delivery to *colonia* residents. This draws out another key question in terms of why these determinants—self-efficacy, *promotores*' certification status, intention to implement the intervention, gender, educational level, and work status—were not associated in this study with the delivery of an intervention to the priority population. Future research can examine these and other psychosocial determinants to further elucidate possible associations. For example, *promotora* marital status and work experience in occupations may be important psychosocial determinants influencing *promotores*' ability to voluntarily deliver cancer education interventions. Additionally, the regression models showed variance in the models, yet the psychosocial determinants were not significant in explaining this variance. This finding brings additional questions as to what else could explain the variance in the models if not the independent variables. This suggests that additional psychosocial determinants may need to be examined and also a larger sample size could yield significant results for these same psychosocial determinants. In this regard, the small sample size could be prone to Type II error. The results from this study demonstrate the need for future studies to continue investigating what psychosocial determinants of *promotores* do influence delivery of interventions since this is one of the first studies to do so.

### Challenges and Limitations

The lack of a control group as well as the exploratory nature of this pre-post, one group design, quasi-experimental study limits this study's demonstration of causation. While comparisons of pre- and post-self-efficacy involve panel data permit inferences of causation, as does the implementation of time-lagged independent and dependent variables in the regression analysis, the study does not prove causation, for which further study is warranted to examine potential causal links. In addition, the results of this study may not be generalizable to all Hispanic groups and *promotores* because there may be significant variations among both *promotores* and Hispanic residents since the samples were not randomly selected or assigned to intervention condition. Another limitation was that the study did not use a study size calculation and also used a convenience sample so whether or not a randomized design would yield different or identical results is unclear. Further, response to study questions may have been influenced by subject's educational level or other factors and skew study results. For example, participants with lower educational and literacy levels may not have understood a question on the instruments and could have selected responses that did not accurately reflect their true responses—particularly if participants were not familiar with Likert-type responses. To address this limitation, pre- and post-instruments with third grade reading levels were used. Additionally, the instruments were read aloud to participants with time for them to select responses independently.

Further, the self-efficacy measurement was simplistic and a limitation. Future work can address this limitation through utilization of established markers (for example, “I am confident I can deliver breast cancer prevention information to Hispanics with lower literacy levels within the next 30 days.”). Another potential limitation is not controlling for promotora experience as well addressing employment status of the promotores. Future studies should consider how to measure and control for experience and type of employment of promotores. Non-response and missing data were also a potential limitation, which was handled by replacing missing values with mean scores given the low percentage of missing data. Lastly, another limitation was the small sample size, which could have contributed to type I error.

### Conclusions

The major findings in this study point to the potential connection between promotores' psychosocial determinants and their influence on behavioral outcomes of program participants—which has been studied very little in the past. Knowing what psychosocial determinants that potentially improve behavioral and health outcomes of those served by promotores could help in the recruitment and training of promotores, which in turn, could yield greater health outcomes for the priority populations served by promotores. This study has a number of areas to expand upon. For instance, future studies should examine which psychosocial determinants of *promotores* may influence the delivery of an intervention to the priority population based on the specific type of cancer. Further, studies could explore additional psychosocial determinants and characteristics of *promotores* that might influence the delivery of an intervention that were not examined in this study such as acculturation, social capital, social support, health status, and relationships with the priority community. Also, additional inquiry is needed on how *promotores*' self-efficacy may influence subsequent indicators of resident training.

One strength of the study was the large number of *promotores* trained on the same cancer education interventions and then following them over time to assess the use of these interventions in practice. An additional strength of the study was the use of reliable measures to assess increases in self-efficacy to deliver cancer education interventions. Lastly, another strength of the study was the ability to connect psychosocial measures of *promotores* to examine the scope of their work—the number of Hispanic *colonia* residents who received the cancer education intervention delivered by *promotores*—and to contribute to this literature on which there is currently little information. Our findings are just a starting point for further research on which psychosocial determinants influence delivery of an intervention. Future research should focus on the T3 step of translational science—the identification of new questions (e.g., additional psychosocial determinants of *promotores* that might influence intervention delivery), barriers, and gaps through dissemination and implementation research. This is an iterative process that allows researchers to return to prior translational stages. Once goals are reached in the T3 step, additional studies can then engage in the T4 step of policy research. This might include looking at developing and implementing policies regarding the utilization of *promotores* in intervention in terms of *promotores*' psychosocial determinants.

In closing, this study examined which psychosocial determinants of *promotores* may influence the delivery of the intervention to the priority population based on the specific type of cancer and has numerous possibilities for areas of future research that could significantly impact research practice and design of cancer education interventions delivered by *promotores*.

## Data Availability Statement

The datasets presented in this article are not readily available because the corresponding author conducted the research at a previous institution. Requests to access the datasets should be directed to https://nchwtc.tamhsc.edu/, chw-training@tamhsc.edu.

## Ethics Statement

The studies involving human participants were reviewed and approved by Committee for the Protection of Human Subjects (CPHS), University of Texas Health Science Center; Institutional Review Board (IRB), Texas A&M University. Written informed consent for participation was not required for this study in accordance with the national legislation and the institutional requirements.

## Author's Note

This manuscript is part of a doctoral dissertation. St. John (2014). Psychosocial determinants of promotores and selected outcomes for a cancer education intervention implemented in South Texas colonias. Three-paper dissertation, ProQuest Dissertations, UMI Dissertation Publishing, http://www.proquest.com.

## Author Contributions

JS was the PI on the original EPICO study and conducted this study as part of a dissertation. BR served as the dissertation committee chair and guided the study development, conduction, and analysis. She participated significantly to the writing and revising of the manuscript. HB and MV-S served on the dissertation committee and guided the study development, conduction, and analysis. They reviewed and revised the manuscript. CB was a Co-I on the original study and served on the dissertation committee. He played a significant role in analyzing the data and writing up the results. All authors contributed to the article and approved the submitted version.

## Funding

This project was funded by the Cancer and Prevention Research Institute of Texas (# PP110241).

## Conflict of Interest

The authors declare that the research was conducted in the absence of any commercial or financial relationships that could be construed as a potential conflict of interest.

## Publisher's Note

All claims expressed in this article are solely those of the authors and do not necessarily represent those of their affiliated organizations, or those of the publisher, the editors and the reviewers. Any product that may be evaluated in this article, or claim that may be made by its manufacturer, is not guaranteed or endorsed by the publisher.
